# SARS-CoV-2 infection increases risk of intracranial hemorrhage

**DOI:** 10.3389/fnhum.2022.991382

**Published:** 2022-11-24

**Authors:** Zuhair Hawsawi, Dilaware Khan, Igor Fischer, Jan Frederick Cornelius, Daniel Hänggi, Sajjad Muhammad

**Affiliations:** ^1^Department of Neurosurgery, King Abdulaziz Hospital, Mecca, Saudi Arabia; ^2^Department of Neurosurgery, Medical Faculty, Heinrich-Heine-University, Düsseldorf, Germany; ^3^Department of Neurosurgery, Helsinki University Hospital, Helsinki, Finland

**Keywords:** COVID-19, SARS-CoV-2, intracranial hemorrhage, stroke, clinical neurosurgery

## Abstract

**Introduction:**

SARS-CoV-2 virus infection causes a dysbalanced and severe inflammatory response, including hypercytokinemia and immunodepression. Systemic inflammation triggered by a viral infection can potentially cause vascular damage, which may lead to cardiovascular and neurovascular events.

**Research question:**

The aim was to investigate whether CNS complications are related to COVID-19.

**Materials and methods:**

We examined 21 patients suffering from stroke and intracranial hemorrhage (ICH) and 9 (43%) of them were male. We compared relative frequencies using Fisher’s exact test. As we had few observations and many variables, we used principal component analysis (PCA) to reduce data dimensionality. We trained a linear support vector machine (SVM) on the first two PCs of the laboratory data to predict COVID-19.

**Results:**

Patients suffering from stroke had either hypertension or SARS-CoV-2 infection, but seldom both (OR = 0.05, *p* = 0.0075). The presence of SARS-CoV-2 infection was strongly associated with the logarithm of CRP (*p* = 1.4e–07) and with D-DIMER (*p* = 1.6e–05) and moderately with PT (*p* = 0.0024). SARS-CoV-2 infection was not related to any other factor. CRP, D-DIMER, PT, and INR were all related to each other (*R*^2^ ranging from 0.19 to 0.52, *p* ranging from 0.012 to < 0.0001). The first two PCs covered 96% of the variance in the four variables. Using them, perfect linear discrimination between patients suffering from COVID-19 and other patients could be achieved.

**Discussion and conclusion:**

SARS-CoV-2 infection causes systemic inflammation, which is suggested as a predictor of the severe course of ICH. SARS-CoV-2 infection is an additional risk factor for vascular complications.

## Introduction

The number of SARS-CoV-2 virus-infected patients is increasing rapidly. To this date, this virus has infected more than 100 million people leading to more than 2 million deaths (Johns Hopkins Coronavirus Resource Center). Preventive measures mainly vaccination is the main hope to overcome this pandemic. However, there is still uncertainty regarding the effectiveness of the currently available vaccination. Foremost, long-term outcomes after acute SARS-CoV-2 infection are unknown. In addition, more evidence regarding severe COVID-19 symptoms is not restricted to respiratory system failure, and also little is known about those “alongside” causalities. Our clinical correlative study provides new evidence for the association of COVID-19 to a hither comparatively unreported severe clinical complication, namely, bleeding of intracranial blood vessels. Our work further supports previous observations of others, such as endothelial cell infection and endotheliitis in COVID-19 patients (Varga et al., 2020), SARS-CoV-2 infected patients associated with stroke (Dhamoon et al., 2021), intracerebral hemorrhage (Cheruiyot et al., 2021), and CNS vasculitis, as well as our own previous experiences of coexisting SARS-CoV-2 infection and subarachnoid hemorrhage (Muhammad et al., 2020). This study further warns to monitor SARS-CoV-2 infected people for possibly the early onset of intracerebral bleeding and indicates the need to deeper investigate the interaction of the virus with the vascular system.

## Materials and methods

### Patient recruitment

We performed a retrospective study on the patients admitted with neurological symptoms of a stroke. Clinical, radiological, and biochemical parameters and the SARS-CoV2 infection status (PCR-based) were documented. In addition to SARS-CoV2 infection status, hypertension was investigated as the probable cause of intracerebral hemorrhage. Other comorbidities and possible causes of cerebral hemorrhage, including diabetes, previous stroke, smoking, atrial fibrillation, and ischemic heart disease, were documented (Table 1). Clinical and on-admission laboratory data were collected retrospectively from a single center (Department of Neurosurgery, King Abdulaziz Hospital, Saudi Arabia) during April–June 2020. According to local law, no specific ethical approval to conduct this retrospective study was required. All required ethical and legal formalities according to the Saudi-Arabian national guidelines to conduct this retrospective study have been fulfilled for this work, and are in concordance with international standards such as the declaration of Helsinki.

**TABLE 1:** Demographics, comorbidities, and outcomes.

		COVID-19 positive	COVID-19 negative
No. Of Patients		11	10
Demographics	Age (years)	45 ± 17	58 ± 10
	Male (%)	63%	20%
	Female (%)	37%	80%
Comorbidities	Patients with hypertension	27%	90%
	Patients with diabetes mellitus	36%	20%
	Patients with ischemic heart disease	18%	10%
	Atrial fibrillation	0%	10%
	Carotid stenosis	18%	10%
	Smoking	9%	30%
	Previous stroke/TIA	9%	30%
Outcomes	Death	27%	10%
	Ventilation dependent	81%	10%

### Statistics

Patients’ characteristics and clinical and laboratory data were analyzed using R, version 3.6.1 “Action of the Toes.” Collinearities were detected using linear regression. Odds ratios to count data were computed using Fisher’s exact test. Means between different groups were compared using Student’s *t*-test. When needed, variables were logarithmically transformed to more closely approximate normal distribution. The optimal classification boundary between COVID-positive and COVID-negative classes was obtained by training a linear support vector machine (SVM) from R’s e1071 library. A significance level of 0.05 was used.

## Results

### Patient population

Our study cohort presents the characteristics mentioned below.

### Clinical manifestation of neurovascular related complications during COVID-19

**FIGURE 1:**
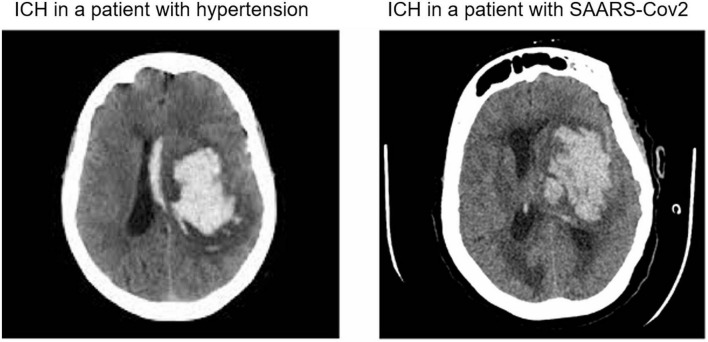
Representative computed tomography (CT) scans of patients with intracranial hemorrhage (ICH) suffering from hypertension or SARS-CoV2 infection/COVID-19.

Intracerebral hemorrhage in patients with COVID-19 infection showed an almost similar pattern of ICH location as in non-COVID-19 infected patients presented with hypertension (Figure 1). However, COVID-19 positive patients presenting with ICH were much younger than the patients with hypertensive bleeding without COVID-19 infection (Table 1). Interestingly, the frequency of male sex in COVID-19 patients with ICH was much higher (63%) than in uninfected patients with ICH (20%). The vascular risk factor, including smoking, arterial fibrillation, and previous TIA, was much less in COVID-19-positive patients with ICH in comparison to uninfected patients with ICH. Diabetes and ischemic heart disease were more frequent in COVID-19-infected patients with ICH. An expected combination of COVID-19 infection and ICH lead to a very high rate of intubation (81%) and mortality of around 27% (Table 1). The presence of COVID-19 infection was strongly associated with the logarithm of CRP (*p* = 1.4e–07), D-DIMER (*p* = 1.6e–05), and moderately with PT (*p* = 0.0024). COVID-19 infection was not related to monocytes, WBC count, neutrophil count, platelet count, blood urea nitrogen, creatinine, alanine transaminase, APTT, or PT. CRP, D-DIMER, PT, and INR were all related to each other, suggesting related pathophysiological mechanisms behind them (*R*^2^ ranging from 0.19 to 0.52, *p* ranging from 0.012 to < 0.0001) (Table 2).

**TABLE 2:** Lab test values.

	COVID-19 positive	COVID-19 negative
Monocytes	0.8 ± 0.5 (*10^9^/L)	0.8 ± 0.4 (*10^9^/L)
WBC count	12.5 ± 4 (*10^9^/L)	12.2 ± 5.2 (*10^9^/L)
Neutrophil count	10.9 ± 3.6 (*10^9^/L)	9.3 ± 5 (*10^9^/L)
Lymphocyte count	1 ± 0.4 (*10^9^/L)	1.5 ± 0.4 (*10^9^/L)
Platelet count	229 ± 74 (*10^3^/mL)	263 ± 117 (*10^3^/mL)
C-reactive protein	30 ± 11 mg/L	6.2 ± 3.6 mg/L
Blood urea nitrogen	10 ± 7.6 mg/dL	8.8 ± 7.3 mg/dL
Creatinine	169 ± 257 mg/dL	84 ± 30 mg/dL
Alanine transaminase (ALT)	34 ± 34 (U/L)	35 ± 35 (U/L)
Aspartate transaminase (AST)	26.5 ± 9.6 (U/L)	59 ± 53 (U/L)
D-DIMER	1.8 ± 0.6 (ng/mL)	0.4 ± 0.1 (ng/mL)
APTT	32.5 ± 6.5 (U/kg/h)	37.4 ± 7.8 (U/kg/h)
PT	14.5 ± 1.9	13.1 ± 1.8
INR	1.3 ± 0.1	1.1 ± 0.2

**FIGURE 2:**
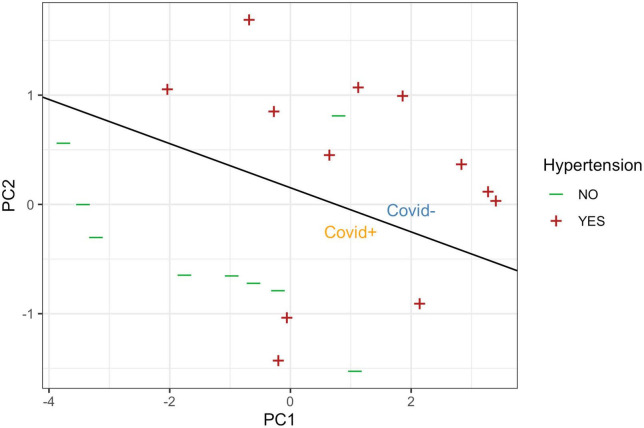
Presence of hypertension, plotted against the first two principal components of the four serum markers (log-CRP, D-DIMER, PT, and INR). The black line denotes the linear class boundary separating the SARS-CoV2/COVID-positive (orange, below) and SARS-CoV2/COVID-negative cases (blue area above the line).

Around 90% of the non-COVID-19 patients with ICH had hypertension as a risk factor. However, COVID-19 positive cohort was mostly non-hypertensive (Table 1). Patients who suffered from a brain hemorrhage had either hypertension or COVID-19 and seldom both (OR = 0.05, *p* = 0.0075) (Figure 2). The first two PCs covered 96% of the variance in the four variables. Using them as predictors, perfect linear discrimination between patients suffering from COVID-19 and other patients could be achieved. Since hypertension is known to be a risk factor for ICH, we may conclude that COVID-19 is also an independent risk factor for ICH.

## Discussion

The SARS-CoV-2 virus uses the angiotensin-converting enzyme 2 (ACE2) receptor, which is highly expressed in the epithelial cells of the lung. As the SARS-CoV-2 virus binds to the ACE2 receptor, the activity of the alternative anti-inflammatory RAS pathway resulting from ACE2-angiotensin interaction is diminished and the classical RAS pathway dominates leading to inflammation, vasoconstriction, water retention, and increased ROS, which consequently can increase the risk of aneurysm rupture due to the hypertensive and ischemic effects of COVID-19 (Panther and Lucke-Wold, 2022; Small et al., 2022). Moreover, three other mediators, including extracellular matrix metalloproteinase inducer (CD147) (Chen et al., 2005), sialic acid (Tortorici et al., 2019), and transmembrane serine protease 2 (Matsuyama et al., 2020), have been suggested to mediate the cellular entry of the virus. The above-mentioned three mediators as well as ACE2 are present in endothelial cells from small and larger arteries and veins, and arterial smooth muscle cells (Hamming et al., 2004). The presence of SARS-Cov-2 elements in endothelial cells and the accumulation of inflammatory cells accompanied by endothelial and inflammatory cell death has been reported (Varga et al., 2020). This damage to endothelial cells by increasing the expression of tissue factors can lead to clotting cascade activation resulting in clot formation, which can lead to stroke when lodged in brain vessels, and these clots *via* partially blocking vessels can cause turbulent flow that can subsequently lead to aneurysm rupture (Panther and Lucke-Wold, 2022; Small et al., 2022). It is already known that cytokine storm (hypercytokinemia) resulting in increased systemic inflammation with high levels of IL-6, IL-1β, and TNFα is induced by viral infections, including influenza A and COVID-19 (Muhammad et al., 2011; Qin et al., 2020). The elevated systemic inflammatory status in COVID-19 patients *via* increasing blood brain barrier permeability can increase MMP-9 and MMP-9 by the dysregulated breakdown of arterial collagen can lead to arterial instability and can consequently result in ICH and aneurysm aSAH (Panther and Lucke-Wold, 2022). All this basic science work clearly provides a mechanistic explanation for SARS-Cov-2 to possibly involve in stroke or stroke-like diseases.

Since the outbreak of the COVID-19 pandemic, some case reports have been published, which suggest the involvement of SARS-Cov-2 infection in hemorrhage and ICH disease course. As such, 0.25–0.7% incidence of ICH COVID-19 patients have been reported (Pavlov et al., 2020; Cheruiyot et al., 2021). In a meta-analysis, out of 108,571 COVID-19 infected patients, acute CVD occurred in 1.4% of patients with 87.4% ischemic stroke (87.4%) and 11.6% ICH cases (Nannoni et al., 2020). Extrapolating assumptions, with currently more than 100 million people infected by COVID-19, would add up to 1 million people, who are possibly at a higher risk for COVID-19-related ICH. In concordance, 105 (38%) stroke patients were SARS-Cov-2 positive out of 277 stroke patients (Dhamoon et al., 2021). The virus-infected stroke patients showed worse outcomes and had higher hospital death rates as compared to stroke patients that did not suffer from COVID-19 (Dhamoon et al., 2021). In support, the presented results—which to our knowledge is the first clinical cohort probing COVID-19 and its correlation to ICH/stroke insults—further evidence the new infectious disease to be an independent risk factor for the development of ICH.

We acknowledge that our study has several technical and experimental limitations. First, the data was collected at a single center. Multicenter studies, involving independent clinical units, are desirable to confirm our findings. Second, our results are based on an overall relatively low total number of patients and all of them are derived from the same ethnical background. We believe that the data are solid for drawing our conclusion in the view of the global state-of-the-art clinical research. Third, no information is available on the patient’s status regarding the risky lifestyle known to increase the risk of ICH development, such as alcohol abuse or smoking. It is possible that the COVID-19 patients are higher abusers as compared to the COVID-19-negative group.

Our study warns about the hitherto unrecognized association of COVID-19 neurovascular complications. Since COVID-19 is an emerging disease, our data suggest close monitoring of the patients for signs of developing neuronal bleeding. In consequence, timely preventive measures can be possibly taken to reduce the deadly secondary effects of the viral infection. Because the systemic inflammation index has been suggested as a predictor of the severity of the course of the disease (Muhammad et al., 2021), the correlation of the inflammatory index, radiology data, and COVID-19 symptoms will help to get more accurate and timely diagnosis. It will further establish the association of the viral infection with neurovascular rupture. In addition, post-mortem studies looking into the brain vasculature will provide more evidence in the support of SARS-Cov-2 to promote hemorrhage diseases.

## Conclusion

Our correlative analysis indicates that SARS-Cov-2 infection increases the risk to develop ICH. Further studies with larger patients population derived from multi-center cohorts, ideally including ethnically diverse cases as well as the inclusion of more detailed patient history data, such as alcohol abuse or smoking habits, are required to validate our hypothesis.

## Data availability statement

The original contributions presented in this study are included in the article/supplementary material, further inquiries can be directed to the corresponding author.

## Ethics statement

Ethical review and approval was not required for the study on human participants in accordance with the local legislation and institutional requirements. Written informed consent for participation was not required for this study in accordance with the national legislation and the institutional requirements.

## Author contributions

SM: conception of the work. ZH, DK, and IF: data collection, analysis, and interpretation. DK, JC, DH, and SM: drafting article and critically reviewing. All authors have read and agreed to the published version of the manuscript.
